# Vascular Complications of Cancer Chemotherapy

**DOI:** 10.1016/j.cjca.2015.12.023

**Published:** 2016-07

**Authors:** Alan C. Cameron, Rhian M. Touyz, Ninian N. Lang

**Affiliations:** Institute of Cardiovascular and Medical Sciences, University of Glasgow, Glasgow, United Kingdom

## Abstract

Development of new anticancer drugs has resulted in improved mortality rates and 5-year survival rates in patients with cancer. However, many of the modern chemotherapies are associated with cardiovascular toxicities that increase cardiovascular risk in cancer patients, including hypertension, thrombosis, heart failure, cardiomyopathy, and arrhythmias. These limitations restrict treatment options and might negatively affect the management of cancer. The cardiotoxic effects of older chemotherapeutic drugs such as alkylating agents, antimetabolites, and anticancer antibiotics have been known for a while. The newer agents, such as the antiangiogenic drugs that inhibit vascular endothelial growth factor signalling are also associated with cardiovascular pathology, especially hypertension, thromboembolism, myocardial infarction, and proteinuria. Exact mechanisms by which vascular endothelial growth factor inhibitors cause these complications are unclear but impaired endothelial function, vascular and renal damage, oxidative stress, and thrombosis might be important. With increasing use of modern chemotherapies and prolonged survival of cancer patients, the incidence of cardiovascular disease in this patient population will continue to increase. Accordingly, careful assessment and management of cardiovascular risk factors in cancer patients by oncologists and cardiologists working together is essential for optimal care so that prolonged cancer survival is not at the expense of increased cardiovascular events.

## Résumé

La mise au point de nouveaux médicaments anticancéreux a permis de réduire le taux de mortalité et d’améliorer le taux de survie après 5 ans des patients atteints de cancer. Cependant, nombre de ces nouveaux anticancéreux sont associés à une toxicité cardiovasculaire qui accroît le risque cardiovasculaire de ces patients, notamment en ce qui a trait à l’hypertension, à la thrombose, à l’insuffisance cardiaque, à la cardiomyopathie et à l’arythmie. Cette problématique limite les choix de traitement et peut avoir une incidence négative sur la prise en charge du cancer. La cardiotoxicité des anticancéreux plus anciens comme les agents alkylants, les antimétabolites et les antibiotiques anticancéreux est connue depuis assez longtemps. Les nouveaux agents comme les antiangiogéniques, qui inhibent l’expression de facteurs de croissance endothéliale vasculaire, sont également associés à des pathologies cardiovasculaires, plus particulièrement à l’hypertension, à la thromboembolie, à l’infarctus du myocarde et à la protéinurie. Le mécanisme causal exact des complications liées aux antiangiogéniques demeure encore inexpliqué, mais la dysfonction endothéliale, les dommages vasculaires et rénaux, le stress oxydatif et la thrombose pourraient être des facteurs importants. Le recours de plus en plus fréquent aux nouvelles chimiothérapies et la prolongation de la survie des patients feront encore augmenter l’incidence des maladies cardiovasculaires dans cette population. Les oncologues devront donc travailler de pair avec les cardiologues afin de soigneusement évaluer et prendre en charge les facteurs de risque cardiovasculaire pour assurer les meilleurs soins possibles et ainsi éviter que la prolongation de la survie des patients se fasse au prix d’un nombre accru d’événements cardiovasculaires.

Advancements in the treatment of cancer have improved the prognosis of patients with a wide range of malignancies,^1^ to the extent that treatment is now often given with curative intent.^2^ In tandem with the improved survival from cancer, there has been increasing focus on cardiovascular actions of chemotherapeutic agents. In addition to acute toxic vascular effects of chemotherapeutic agents, the latent effects of direct and indirect cardiovascular toxicity become more relevant. Patients now frequently survive long enough to allow these effects to manifest and become the prime concern.^3^ It has become increasingly complex to establish a pragmatic balance between effective anticancer therapy while mitigating the risks of cardiovascular complications. As a result, cardio-oncology is rapidly growing as a cardiovascular subspecialty in its own right.

Heart failure and heart muscle toxicity induced by chemotherapy, particularly anthracyclines and HER2 receptor antagonists, have benefited from an expanding recognition and evidence base to inform strategies to mitigate the risk of this potentially devastating complication. However, in contrast, there is a smaller evidence base and mechanistic insight to the vascular complications associated with cancer chemotherapeutics. Many conventional chemotherapy agents, as well as some of the newer anticancer signalling inhibitors and antiangiogenic drugs, predispose patients to cardiovascular side effects including hypertension, acute coronary syndromes, and arterial and venous thrombosis (Table 1).^1^, ^2^

Vascular complications of chemotherapy might occur as a result of ‘off-target’ drug effects or, importantly, as a result of the significant overlap between signalling pathways required for normal vascular function and those required for tumour growth. Vascular toxicity of chemotherapy often reflects endothelial dysfunction, with loss of vasorelaxant effects and suppressed anti-inflammatory and vascular reparative functions. These effects might serve to initiate and further perpetuate the development of hypertension, thrombosis, and atherogenesis. In addition to the procoagulant effect of cancer per se, platelet activity is further enhanced by decreased endothelial nitric oxide (NO) bioavailability.^2^

**Figure 1:**
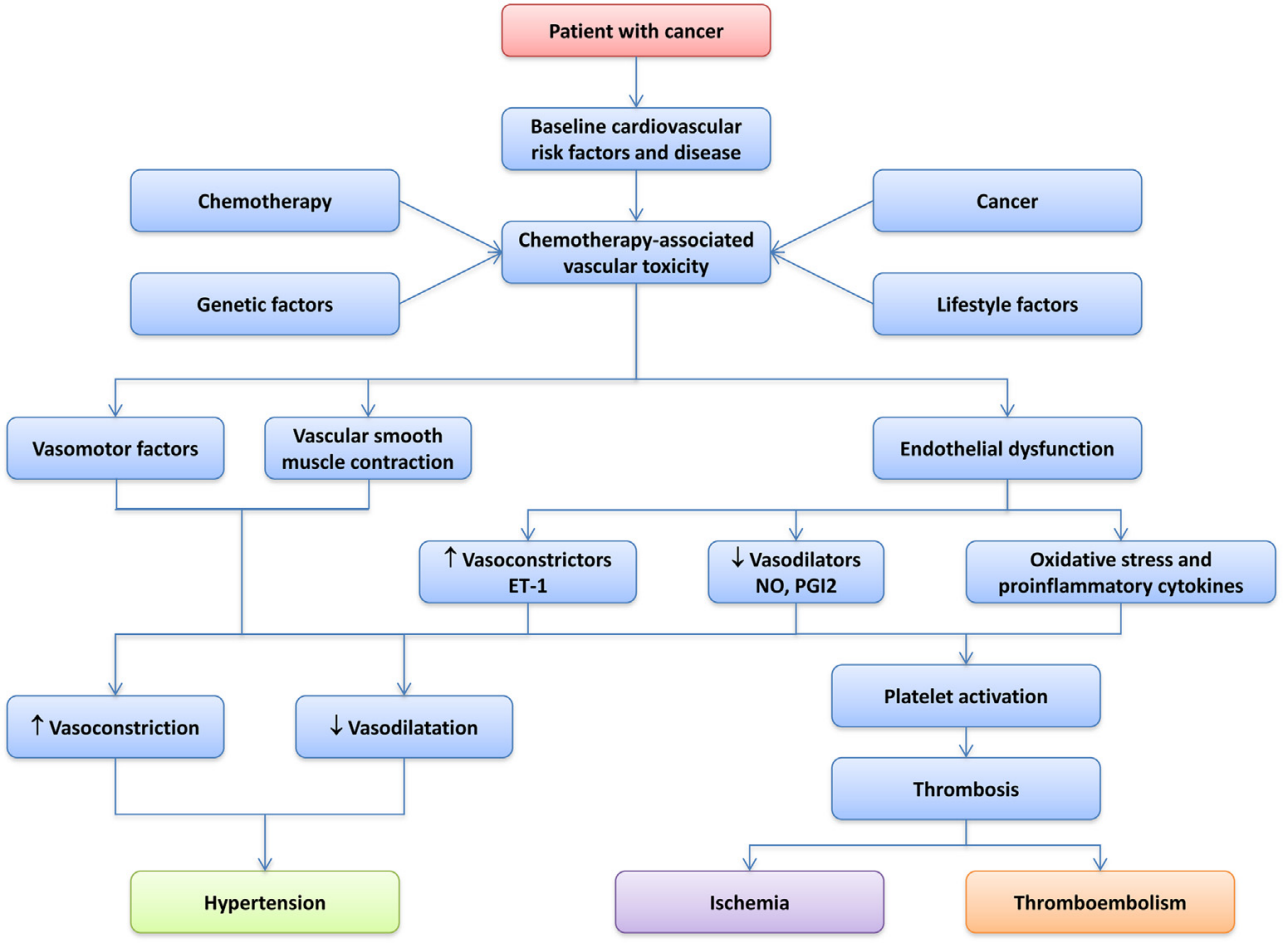
Diagram illustrating factors that possibly contribute to chemotherapy-associated vascular toxicity. Multiple stimuli, such as cardiovascular risk factors, cancer itself, and anticancer drugs, influence vascular function and arterial structure leading to increased reactivity, altered vascular tone, impaired endothelial function, and platelet activation. These processes in turn contribute to cardiovascular disease, such as hypertension, cardiac ischemia and thrombosis, which might be facilitated and aggravated by chemotherapy in cancer patients. ET-1, endothelin-1; NO, nitric oxide; PGI2, prostacyclin.

**Figure 2:**
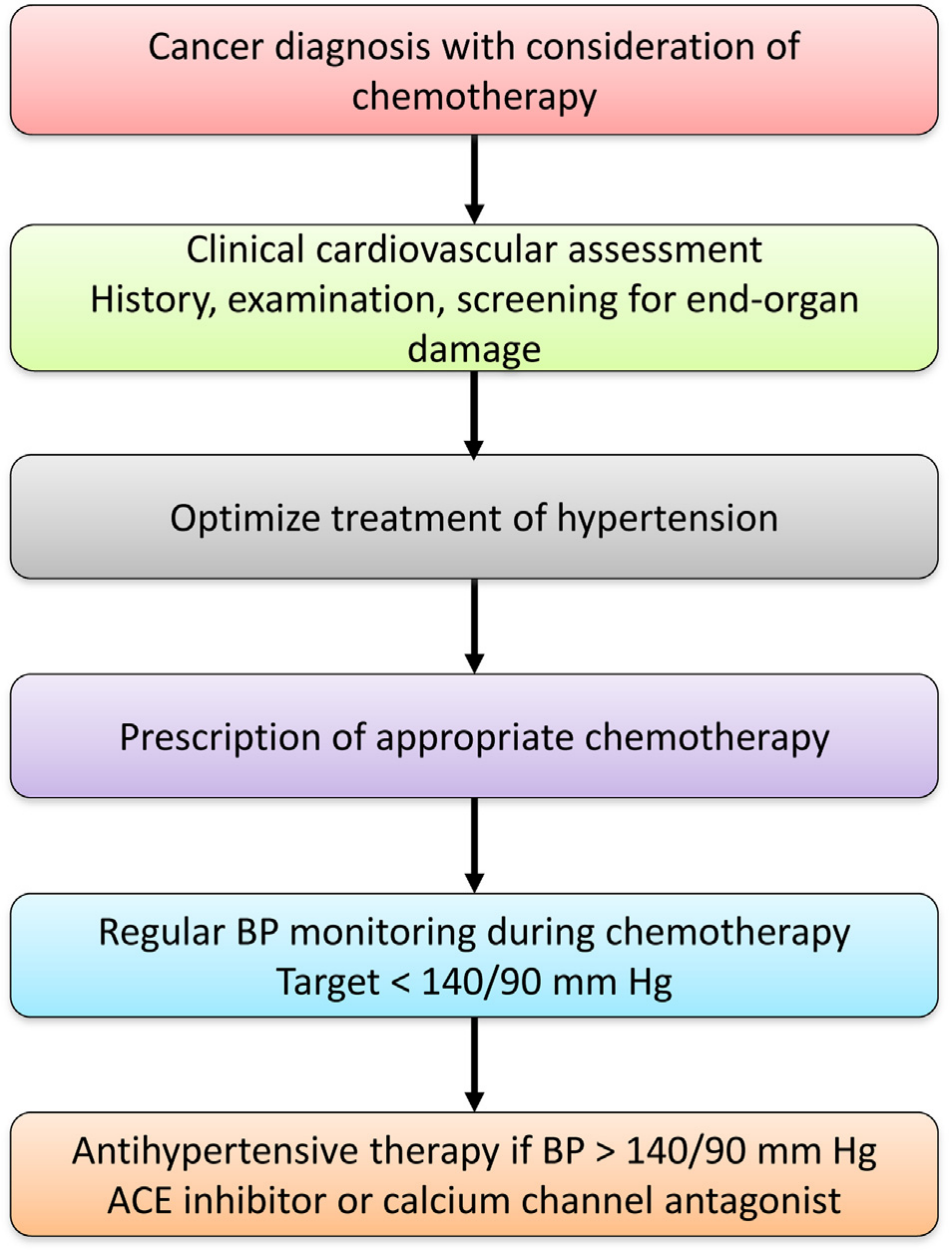
Clinical approach in assessing and managing hypertension in cancer. Flow chart showing clinical approaches to the cardiovascular assessment of patients before and during chemotherapy, and the management of chemotherapy-associated hypertension. ACE, angiotensin-converting enzyme; BP, blood pressure.

The propensity to develop cardiovascular complications of cancer therapy reflects the complex interplay between a patient's baseline cardiovascular risk and preexisting vascular disease, particularly hypertension and diabetes, whilst evidence for genetic predisposition is increasing (Fig. 1).^4^ Optimal strategies for the diagnosis, surveillance, and management of cardiovascular complications in patients who receive chemotherapy agents remain incompletely defined and can be challenging.^1^ Baseline cardiovascular assessment is vital before the selection of appropriate chemotherapy and preexisting cardiovascular disease must be treated aggressively (Fig. 2). Baseline endothelial function is a risk marker for the development of cardiovascular events and correlates well with traditional cardiovascular risk factors in the ‘noncancer’ population. Assessment of endothelial function could therefore be a useful tool for identification of asymptomatic subjects at high cardiovascular risk, and stratification of risk among subjects with known cardiovascular disease before consideration of chemotherapy.^5^ This strategy is not, however, currently used in routine clinical practice.

It might be appropriate to avoid some chemotherapy agents in patients at high risk of vascular complications and in others the potential for vascular toxicity might be safely managed without affecting the net benefit from chemotherapy.^2^ Careful stratification of such patients might lead to reduced cardiovascular morbidity in cancer patients who receive chemotherapy. Indeed, distilling the relative likelihood of vascular toxicity against the anticancer effects of chemotherapy remains an enormous challenge and highlights the absolute requirement for close collaboration between oncologists and cardiovascular specialists. Ongoing and targeted vascular evaluation during treatment is important, often with the early introduction of secondary preventive treatments that might need to be used in a context outwith the evidence base derived from major cardiovascular trials. There remains an unmet need to better stratify patients who might require much longer follow-up or preventative vascular therapies, potentially for years after cancer treatments have ended.

In this review we provide a clinically relevant overview of vascular toxicity associated with major classes of chemotherapy drugs. Although a large body of work addresses significant problems associated with drug-induced heart muscle toxicity and heart failure,^1^, ^4^ we focus on the vascular effects, particularly hypertension, and arterial and venous thrombosis.

## Vascular Endothelial Growth Factor Inhibitors

Angiogenesis, the process of new blood vessel formation, is central to solid tumour growth and metastasis,^6^, ^7^ and is therefore an ideal target for anticancer therapy. Vascular endothelial growth factor (VEGF) is among the most important of growth factors involved in angiogenesis. VEGF is a 45-kDa glycoprotein produced by many cell types, including endothelial progenitor cells, endothelial cells, renal epithelial cells, fibroblasts, macrophages, and certain tumours.^8^ The *VEGF* gene undergoes alternative splicing to form multiple isoforms: VEGF-A, VEGF-B, VEGF-C, VEGF-D, and placental growth factor. VEGF-A, the best characterized, binds to 3 types of tyrosine kinase receptors (VEGF receptor [VEGFR]1, VEGFR2, and VEGFR3).^8^, ^9^ VEGFR1 and VEGFR2 are expressed predominantly in endothelial cells, with VEGF-A binding to VEGFR2 having the major vascular effects. Activation of VEGFR2 by ligand binding initiates signalling through tyrosine kinases that stimulate many pathways, including phosphoinositide 3-kinase/AKT/protein kinase B-mammalian target of rapamycin, endothelial NO synthase, and prostacyclin, that regulate vasodilation and inflammatory responses.^10^, ^11^ VEGF also signals through phospholipase C, Raf-1, and mitogen-activated protein kinases, pathways that regulate endothelial cell survival, proliferation, migration, and permeability.^12^

Chemotherapy agents might influence VEGF effects directly, as is the case for VEGF inhibitors (VEGFIs), or as a secondary effect as occurs with the ‘classical’ cytotoxic drugs, including antimetabolites, taxanes, anthracyclines, and alkylating agents.^5^, ^13^, ^14^ Interruption of VEGF signalling is associated with the development of vascular toxicity and clinical sequelae such as hypertension, acute coronary syndromes, stroke, venous thrombosis, and thromboembolism.^5^, ^15^, ^16^, ^17^, ^18^

VEGFIs are now the cornerstone of therapy for a wide variety of solid tumours and hematological malignancies. There are 3 main groups of VEGFIs: (1) monoclonal antibodies against circulating VEGF (eg, bevacizumab [Avastin; Roche]). This class of agent selectively binds to circulating VEGF to inhibit its interaction with the VEGFR. As such, it is considered a relatively specific VEGF signalling pathway antagonist; (2) small-molecule inhibitors of intracellular tyrosine kinases (eg, sunitinib [Sutent; Pfizer]), sorafenib [Nexavar; Bayer]). These agents are not VEGFR2-specific and also inhibit other receptor tyrosine kinases, including platelet-derived growth factor and c-Kit signalling, which are implicated in tumour angiogenesis.^19^ This combined effect increases antiangiogenic and anticancer efficacy but also contributes to a greater risk of adverse cardiovascular effects^19^, ^20^, ^21^, ^22^, ^23^; and (3) VEGF ‘trap’ (eg, aflibercept [Zaltrap; Sanofi]); this recombinant fusion protein comprises VEGF-binding regions of VEGFRs 1 and 2.^2^, ^24^

### Hypertension

Hypertension is the most common cardiovascular complication associated with VEGFIs.^7^ Almost all patients have an absolute increase in blood pressure with most clinical trials reporting increased blood pressure as an adverse effect and up to 80% of patients developing hypertension that is often severe and difficult to treat.^7^, ^25^, ^26^, ^27^ The true prevalence of VEGFI-induced hypertension might be underestimated because of varying definitions used in clinical trials with blood pressure thresholds often greater than those used in most evidence-based guidelines.^7^, ^28^ Furthermore, patients with difficult to treat hypertension or a history of cardiovascular disease are usually excluded from clinical trials.^7^ There have been recent efforts to aid consistency in the diagnosis and reporting of VEGFI-associated hypertension, which should follow the 2014 Joint National Commission recommendations, with a threshold of 140/90 mm Hg on 3 occasions at least 1 week apart in individuals aged younger than 60 years and 150/90 mm Hg in those aged older than 60 years.^7^, ^29^, ^30^

The exact mechanisms of VEGFI-induced hypertension are not fully understood but are believed to include endothelial dysfunction, reduced NO generation and vasodilatation, increased endothelin-1 and vasoconstriction, capillary rarefaction, vascular remodelling, and oxidative stress.^7^, ^31^, ^32^, ^33^, ^34^, ^35^, ^36^ Recent data from animal models and early clinical studies have suggested autonomic nervous system toxicity might also contribute to VEGFI-associated hypertension.^37^ An acute, dose-dependent increase in blood pressure occurs within hours to days of starting treatment and resolves rapidly upon withdrawal of the agent.^7^, ^38^ Patients with a history of hypertension and those treated with multiple VEGFIs are at particularly high risk.^7^, ^32^, ^33^, ^34^, ^35^, ^39^

All patients should undergo a comprehensive assessment screening for existing cardiovascular disease before treatment with a VEGFI. This should include a history, physical examination, and screening for end-organ damage (Fig. 2). Thereafter, there should be active monitoring of blood pressure during treatment, particularly in the first cycle when blood pressure should be checked weekly, followed by every 2-3 weeks. Increased blood pressure should be treated aggressively, aiming for a target of < 140/90 mm Hg.^7^, ^29^, ^40^, ^41^ Angiotensin-converting enzyme inhibitors and dihydropyridine calcium channel antagonists appear most effective in the treatment of VEGFI-induced hypertension (Table 2), although the evidence from which to draw these conclusions remains relatively small. Thiazide diuretics, mineralocorticoid receptor antagonists and β-blockers may be used if additional antihypertensive agents are required.^40^, ^42^, ^43^ Angiotensin-converting enzyme inhibitors also have the potential to protect against proteinuria and direct cardiac toxicity associated with chemotherapy. Mineralocorticoid receptor antagonists are increasingly being used to treat resistant hypertension^43^ and might therefore have a role in the management of VEGFI-associated hypertension.

Nondihydropyridine calcium channel antagonists, such as verapamil or diltiazem, should be avoided because these agents inhibit cytochrome P450 3A4, through which VEGFIs are metabolized. The coprescription of verapamil or diltiazem can provoke increased plasma antiangiogenic drug concentrations.^7^ Care should also be taken to note potential ‘off periods’ in VEGFI regimens to avoid symptomatic rebound hypotension.^7^

The use of NO donors or endothelin receptor antagonists in the treatment of VEGFI-associated hypertension has yet to be formally assessed in a rigourous trial. However, this iatrogenic cause of hypertension might provide a ‘niche’ role for endothelin receptor antagonists and preclinical data and mechanistic insight provide enthusiasm for this approach.^7^

### Thrombosis

Although VEGFIs have been associated with thrombotic and hemorrhagic side effects,^44^, ^45^ the prothrombotic effects appear to predominate. VEGFIs are associated with an absolute increase in risk of arterial and venous thrombosis and thromboembolism of 1.5%-4%. The risk of arterial thrombosis appears to be greater than that of venous thrombosis.^4^, ^46^, ^47^, ^48^

Bevacizumab is associated with a 2.1-fold increased risk of high-grade cardiac ischemia^49^; sorafenib was associated with a 3% incidence of myocardial ischemia or infarction,^50^ and a study of sunitinib in patients with advanced clear-cell renal carcinoma showed a 1% incidence of myocardial infarction (MI).^20^

Faruque and colleagues showed a 3.5-fold increased risk of MI and 1.8-fold increased risk of arterial thrombosis associated with VEGFI therapy.^51^ However, the absolute increase in risk is relatively small (0.8% and 1.8% increased risk for MI and arterial thrombosis, respectively), but clinically important, particularly for those with preexisting risk factors or vascular disease. Patients with previous coronary artery disease are at particularly high risk of developing vascular complications^4^, ^52^ and it might be reasonable to consider screening patients for preexisting coronary artery disease before commencing antiangiogenic treatment, although the most appropriate method remains unclear.^4^ Because the pathophysiologic mechanism partly reflects increased platelet activation, as a consequence of endothelial dysfunction, there might be a role for antiplatelet therapy but concerns over coexisting propensity to hemorrhage have limited the applicability of this strategy. Insufficient data are available to clearly guide the optimum preventative therapy,^4^ and the potential cardioprotective effects of statin therapy in this context also remains to be evaluated.^4^

## Tyrosine Kinase Inhibitors for Hematologic Malignancy

Tyrosine kinase inhibitors (TKIs) developed for use in the treatment of hematologic malignancy, including ponatinib, nilotinib, and dasatinib, are associated with a particularly high incidence of acute arterial thrombosis. This is particularly evident for ponatinib,^4^ which acts as a potent multitargeted TKI against the oncogenic fusion gene, *Bcr-Abl*, and is used in the treatment of chronic myeloid leukemia (CML) and Philadelphia chromosome-positive acute lymphoblastic leukemia resistant to, or intolerant of, traditional TKIs.^4^ Its efficacy was shown in the Ponatinib for CML Evaluation and Philadelphia Chromosome-Positive Acute Lymphoblastic Leukemia trial, in which it had significant antileukemic action across both groups of patients and categories of disease stage.^53^ It was, however, associated with an almost 12% incidence of arterial thrombotic events at 2 years, with most events occurring as an acute thrombotic process.^4^

Ponatinib is also associated with relatively high rates of venous thrombosis, with an incidence of 2.2% at 1 year and 2.9% at 2 years. Although this association is clinically relevant, the incidence of venous thrombosis is markedly lower than that of arterial thrombosis. The high rates of vascular events initially led to the withdrawal of ponatinib, although it was reintroduced in 2014 with a US Food and Drug Administration ‘black box’ warning.^4^

Nilotinib, also an anti-Bcr-Abl TKI, preceded the use of ponatinib in the treatment of CML.^4^ It is also associated with high rates of arterial thrombosis, with 25% of patients experiencing an acute arterial event in the initial study.^4^, ^54^ The 2-year incidence of acute arterial event is almost 15% with a 33% predicted 10-year risk of progressive peripheral arterial disease.^4^, ^55^ The risk appears to remain high regardless of baseline cardiovascular status.^4^, ^56^ Furthermore, there appears to be a predilection for peripheral arterial disease involving the lower limbs, as well as the renal and mesenteric arteries, and attendant vascular complications, including renovascular hypertension and ischemic nephropathy.^4^, ^57^, ^58^

Mechanisms underpinning the high incidence of acute arterial events associated with ponatinib and nilotinib remain unclear, although not all anti-Bcr-Abl TKIs are associated with such high risk. The 10-year projected risk of developing progressive peripheral arterial disease is 14 times greater with nilotinib than with the prototype anti-Bcr-Abl TKI, imatinib.^55^ The reason for such a discrepancy remains unclear and is not accounted for by age, sex, or traditional cardiovascular risk factors.^55^ There might be as yet unidentified off-target effects, as has been observed with TKIs of the VEGF signalling pathway. Because the pathophysiological mechanisms that contribute to the high risk of arterial vascular events remain unclear, the most appropriate approaches to management also remain unclear.^4^

## Alkylating Agents

### Platinum-based compounds

Cisplatin is associated with acute and late cardiovascular side effects, including hypertension, myocardial ischemia and infarction, thromboembolism, and cerebrovascular disease.^2^, ^59^ These effects might contribute to a cardiovascular risk profile more concerning than the risk of cancer relapse or the development of a second malignancy.^5^, ^60^

#### Hypertension

Hypertension is a frequently reported complication of cisplatin-based chemotherapy.^5^, ^60^, ^61^, ^62^, ^63^, ^64^ The prevalence varies with Sagstuen and colleagues reporting that 53% of patients treated with higher dose cisplatin therapy developed hypertension over a median follow-up of 11 years (odds ratio, 2.3; 95% confidence interval, 1.5 to-3.7, compared with control participants).^62^ Other studies reported a prevalence that ranged from 39% of patients at least 10 years after treatment,^59^ to 28% over 7 years,^65^ and 14% over 14 years.^64^ Strumberg and colleagues reported no significant increase in systolic blood pressure after 13 years of follow-up after cisplatin-based chemotherapy for testicular cancer, although 25% of subjects developed diastolic hypertension.^60^ Overall, most studies showed that a significant proportion of patients develop persistent hypertension after cisplatin-based chemotherapy, with endothelial cell activation, damage, and subsequent endothelial dysfunction believed to be the main contributing factor.^5^, ^61^, ^64^, ^66^ Microalbuminuria, a marker of endothelial dysfunction, occurs in up to 22% of patients at least 10 years after cisplatin-based chemotherapy for metastatic testicular cancer.^59^ Calcium channel antagonists appear most effective in the treatment of hypertension associated with alkylating agents such as cisplatin and patients treated with alkylating agents should have long-term monitoring of their cardiovascular risk profile, including blood pressure.

#### Thrombosis

Cisplatin-based chemotherapy is associated with a 9% risk of thromboembolic complications.^5^, ^66^, ^67^ Potential mechanisms that might contribute to thrombus formation include endothelial cell damage and dysfunction provoking a hypercoagulable state with platelet activation, adhesion, and aggregation, increased von Willebrand factor, and reduced NO bioavailability.^2^, ^18^, ^68^ Cerebrovascular complications might occur through thrombosis in situ as a consequence of endothelial dysfunction or by thromboembolism.^5^ Furthermore, cisplatin-associated hypertension might result in acute cardiovascular complications during therapy as well as contribute to the initiation and progression of atherosclerotic cardiovascular complications in the longer-term.^5^

Over a median follow-up of 14 years, cisplatin-based chemotherapy for metastatic testicular cancer has been associated with a sevenfold increased risk of major cardiac event (6% of patients).^59^ Preclinical and clinical data provide convincing evidence that the toxicity reflects primarily the platinum (cisplatin) component of this regimen.^59^, ^69^ Patients previously treated with platinum-containing compounds also show persistent adverse cardiovascular risk profiles, including hypertension and hyperlipidemia.^59^, ^70^ Fung and colleagues recently showed an almost fivefold increased risk of cardiovascular mortality in the first year after testicular cancer diagnosis in patients treated with cisplatin compared with surgery alone.^71^ The risk of thrombotic complications decreased markedly after 1 year. This large study should focus our attention on the short- to medium-term effects of platinum-based chemotherapy and suggests that the acute toxic effects of platinum-based chemotherapy predominate over concerns about an adverse cardiovascular risk profile in the longer-term.

Although endothelial toxic effects of cisplatin-based chemotherapy appear to be central in the pathophysiology of associated complications, abnormalities in endothelial function assessed using measures of brachial artery flow-mediated dilatation have not shown a consistent effect over time. When assessed within 10 weeks of administration of platinum-based chemotherapy,^69^ no change in flow-mediated dilatation was observed although marked decreases were seen immediately after treatment^72^ and also at 1 year.^73^ Therefore, the time course of endothelial impairment remains incompletely defined. It is conceivable that there is a biphasic response with ‘hyperacute’ and partially reversible endothelial toxicity in the immediate peritreatment period followed by a subsequent decline as a result of a persistent adverse cardiovascular risk profile.

Cisplatin also has the potential to provoke vasospasm, which might result in symptoms of angina, acute coronary syndrome, and stroke.^18^ Additionally, cisplatin can provoke hypomagnesemia, which might contribute to arrhythmias, and alterations in vascular tone with coronary and cerebral artery vasospasm.^18^, ^74^

#### Nephrotoxicity

Nephrotoxicity is a well documented adverse effect of cisplatin use, with a dose-dependent and irreversible reduction in renal function.^75^, ^76^, ^77^ Possible mechanisms include endothelial and epithelial cell damage and dysfunction.^78^ Patients treated with cisplatin-based chemotherapy have a high prevalence of microalbuminuria^62^, ^66^ and there are indications to suggest that patients who develop microalbuminuria in association with cisplatin-based chemotherapy have higher blood pressure levels than those who do not.^59^, ^62^ This supports the hypothesis that endothelial dysfunction and associated nephrotoxicity might contribute to hypertension associated with cisplatin-based chemotherapy.^62^

### Cyclophosphamide

This alkylating agent is associated with vascular complications including hypertension, MI, stroke, Raynaud phenomenon, and hepatic veno-occlusion. Circulating concentrations of VEGF are reduced by cyclophosphamide administered at continuous low doses, which might underpin some of the observed vascular toxicity, as seen in patients treated with VEGFIs. Notably, cyclophosphamide is also associated with the development of interstitial pneumonia and pulmonary fibrosis, with lung biopsy showing vascular sclerosis and signs of pulmonary hypertension.^5^ This might be a consequence of reduced angiotensin-converting enzyme activity as well as neutrophil and monocyte adhesion to damaged endothelium with platelet accumulation in endothelial lesions.^5^

## Antimetabolites: 5-Fluorouracil

5-Fluorouracil (5-FU) and its prodrug, capecitabine, are mainly associated with myocardial ischemia, which might be due to primary coronary artery spasm, thrombosis, or endothelial dysfunction.^1^, ^2^, ^18^, ^79^, ^80^ Myocardial ischemia might present as a broad spectrum from asymptomatic ST segment changes on electrocardiogram through to angina, MI, and sudden cardiac death.^1^ Most events occur early and the risk is greatest when administered at high, repeated doses and as a continuous infusion. Although endothelial cell damage and thrombus formation might contribute to myocardial ischemia, coronary artery vasospasm is believed to be the main pathogenic factor.^18^, ^81^, ^82^ 5-FU can exert direct toxic effects on vascular endothelium to reduce endothelial NO synthase activity and provoke coronary artery vasospasm, and endothelium-independent vasoconstriction via protein kinase C.^5^, ^83^ The coronary endothelium is particularly susceptible to these effects, leading to a Prinzmetal-type angina phenomenon.^5^ Although 5-FU exerts acute effects on the coronary arteries in terms of vasospasm and possibly thrombus formation, it has not been associated with the development of accelerated coronary atherosclerosis.^18^

The addition of bevacizumab has a synergistic effect on the vascular complications associated with 5-FU,^5^, ^84^ which is consistent with the hypothesis that cardiovascular effects are mainly related to vasospasm and altered vascular reactivity.^5^ However, experimental studies have also implicated endothelial and myocardial cell apoptosis,^5^ although 5-FU causes a dose-dependent increase in red blood cell viscosity and reduced blood flow velocity that predispose to thrombus formation.

Preexisting coronary artery disease remains a risk factor for 5-FU-related vasospastic angina,^18^, ^85^ which most likely reflects the observation that vasospasm tends to occur at sites of thrombus and plaque formation.^18^ Repeated ‘challenge’ with 5-FU or capecitabine tends to result in recurrent symptoms and alternative agents should be used when toxicity has occurred.

## Anticancer Antibiotics

### Anthracyclines

The anthracyclines, such as doxorubicin and epirubicin, represent some of the most effective chemotherapy agents and are used widely in the treatment of hematological and solid malignancies including breast and gastric cancer, leukemia, and lymphoma.^4^, ^86^ They are also, however, associated with profound cardiotoxicity and have a marked long-term effect on cardiac structure and function. The principle side effect is a cumulative, permanent, and dose-related cardiotoxicity with consequent left ventricular dysfunction and heart failure.^86^, ^87^ This is referred to as type 1 cardiotoxicity, and type 2 cardiotoxicity, which is generally associated with agents such as trastuzumab, is not dose-related and usually resolves with discontinuation of therapy.^3^ Anthracycline-associated cardiotoxicity therefore appears to occur through direct pathophysiological mechanisms rather than as a secondary consequence of systemic hypertension, or arterial or venous thrombosis.^87^, ^88^

### Bleomycin

Bleomycin exerts anticancer effects by damaging DNA and disrupting the cytoskeleton. It causes a dose-dependent reduction in endothelial cell growth and induction of apoptosis. These vascular toxic effects at least in part explain associated cardiovascular complications including myocardial ischemia and infarction, thrombosis and thromboembolism, pulmonary fibrosis, and Raynaud phenomenon.^5^

### Microtubule targeted agents (including taxanes and vinca alkaloids)

Microtubule targeted agents include the taxanes (eg, paclitaxel), and the vinca alkaloids (eg, vincristine and vinblastine).^89^ They act to alter the cellular microtubule mass, which represents one of the most successful targets for chemotherapy agents.^89^

The taxanes have significant antiangiogenic properties and cause disruption of the cytoskeleton and impaired endothelial cell function.^5^, ^90^ At low doses, they block critical signalling pathways and prevent cell motility and cell-cell interactions.^5^, ^91^ At higher doses, they cause microtubule deficiency with endothelial cell detachment and apoptosis.^5^ Paclitaxel attenuates vascular smooth muscle cell migration and halts endothelial cell proliferation.^5^, ^92^ It might also have prothrombotic effects through enhanced endothelial tissue factor expression via selective activation of c-jun kinase.^5^, ^93^ Docetaxel inhibits endothelial function in vitro and angiogenesis in vivo^5^, ^94^ and shows a dose-dependent vascular toxicity, including a “fluid retention syndrome” due to capillary leakage.^5^, ^95^

The vascular side effects of taxanes are compounded when used in combination with angiogenesis inhibitors. The combination of bevacizumab with paclitaxel in patients with advanced breast cancer increases the rate of severe thrombotic events from 1.5% to 2.1%, and the combination of bevacizumab with a paclitaxel-carboplatin combination therapy in patients with advanced non-small-cell lung cancer increases the rate of severe hypertension from 0.7% to 7%.^5^, ^96^, ^97^

The vinca alkaloids, vincristine and vinblastine, are tubulin binders that precipitate cell death and are primarily used in the treatment of leukemia and lymphoma. Their main cardiovascular side effects are myocardial ischemia and infarction, which tend to occur during or shortly after therapy and might therefore be related to coronary artery vasospasm as a result of cellular hypoxia.^18^

## Conclusions

Cardiovascular complications of cancer chemotherapy are common and have increased in parallel with improved cancer survival. This not only reflects the toxicity of classical and novel agents, but also survival long enough after cancer diagnosis for cardiovascular complications, or acceleration of preexisting disease, to become clinically relevant.

The spectrum of vascular complications associated with individual agents needs to be borne in mind when selecting the optimum chemotherapeutic regimen on a patient by patient basis, taking into account background cardiovascular risk and comorbidity. However, the ideal means to assess optimal therapy and likelihood of ‘net benefit’ remains poorly defined. In addition to conventional screening for cardiovascular risk factors it remains to be seen whether noninvasive assessment of endothelial function provides incremental value in risk prediction. This question is particularly relevant because of the central role of the endothelium in the pathophysiological mechanisms underpinning vascular toxicity. A pragmatic approach needs to be taken to balance the most efficacious anticancer treatment with minimal vascular toxicity. Cross-disciplinary management and decision-making for the group of patients who require oncologic and cardiovascular expertise has progressed but there remains room for improvement.

Reporting of vascular complications in clinical trials of cancer therapies requires greater consistency. With the benefit of clearer clinical end points we will be in a position to design better prospective trials to evaluate potential vascular protective strategies for patients who undergo chemotherapy and understand the best treatment of complications when they have occurred. The appropriateness of continuing, reducing, or changing chemotherapy agents and the best way to treat cardiovascular and renal complications when they have occurred is still an area of debate and often little consensus.

Patients have benefitted enormously from advances in cancer and cardiovascular therapies. The challenge now exists to keep pulling these 2 clinical and research disciplines closer together so that investigators in these historically ‘separate’ areas can work collaboratively to ensure that a good oncologic response to treatment does not come at an unacceptable cardiovascular price.

## Funding Sources

Work from the author's laboratory was supported by grants from the British Heart Foundation (BHF; RG/13/7/30099). R.M.T. is supported through a BHF Chair (CH/12/4/29762), and A.C.C. is supported through a Fellowship funded through a BHF Award of Research Excellence to the University of Glasgow.

## Disclosures

The authors have no conflicts of interest to disclose.

## Figures and Tables

**Table 1:** Chemotherapy agents with principal cardiovascular complications and potential mechanisms

Chemotherapy drug class	Chemotherapy agents	Principle cardiovascular complications		Potential mechanisms
VEGF signalling pathway inhibitors				
	Bevacizumab Sunitinib Sorafenib	Hypertension	++++	Endothelial dysfunction ↓ NO signalling ↑ ET signalling Capillary rarefaction Vascular remodelling Oxidative stress
Ischemia Thromboembolism	+	Platelet activation ↓ NO and PGI_2_ signalling
Tyrosine kinase inhibitors for hematological malignancy				
	Ponatinib Nilotinib Dasatinib	Ischemia	++++	Acute arterial thrombosis
Alkylating agents				
	Cisplatin	Hypertension	++++	Endothelial dysfunction
Ischemia Thromboembolism	++	Platelet activation ↓ NO and PGI_2_ signalling Vasospasm
Nephrotoxicity	++++	Endothelial dysfunction
Antimetabolites				
	5-Fluorouracil	Ischemia	++++	Vasospasm
Anthracyclines				
	Doxorubicin Epirubicin	Cardiotoxicity	+++	Myocyte apoptosis

Approximate frequency of complications indicated by + (< 5%), ++ (5%-10%), +++ (10%-20%), and ++++ (> 20%).

ET, endothelin; PGI_2_, prostacyclin; NO, nitric oxide; VEGF, vascular endothelial growth factor.

**Table 2:** Summary of the approaches to management of chemotherapy associated hypertension

Aspect of therapy	Drug class	Examples	Indications/benefits	Cautions/contraindications
First- and second-line therapy	ACE inhibitors	Captopril Enalapril Lisinopril Perindopril Ramipril	• VEGFI associated hypertension • Younger patients • Proteinuria • Diabetic nephropathy • Left ventricular dysfunction • Quick onset of action	• Renovascular disease • Peripheral vascular disease • Renal impairment • Chemotherapy with renal clearance • Hyperkalaemia
Angiotensin II receptor antagonists	Candesartan Irbesartan Losartan Valsartan	• VEGFI-associated hypertension • Cough related to ACE inhibitor • Younger patients • Proteinuria • Diabetic nephropathy • Left ventricular dysfunction • Quick onset of action	• Renovascular disease • Peripheral vascular disease • Renal impairment • Chemotherapy with renal clearance • Hyperkalemia
Dihydropyridine calcium channel antagonists	Amlodipine Lercanidipine	• Cisplatin-associated hypertension • Elderly patients • Isolated systolic hypertension	• Ankle swelling • Slow onset of action
Third-and fourth-line therapy	Thiazide diuretics	Bendroflumethiazide Chlorthalidone Hydrochlorothiazide Indapamide	• Elderly patients • Isolated systolic hypertension	• Gout • Hypercalcaemia • Hypokalaemia • QTc prolonging drugs
Mineralocorticoid receptor antagonists	Eplerenone Spironolactone	• Resistant hypertension	• Hyperkalemia • Gynecomastia (spironolactone)
β-blockers	Bisoprolol Carvedilol Metoprolol	• Ischemic heart disease	• Bradycardia • Heart block • Asthma or COPD
Agents to avoid	Non-dihydropyridine calcium channel antagonists	Verapamil Diltiazem	N/A	
BP management during chemotherapy “off periods” or after stopping or completing chemotherapy	• Monitor for rebound hypotension with or without downtitration or stop antihypertensive therapy • Regular monitoring of blood pressure after stopping or completing chemotherapy			

ACE, angiotensin-converting enzyme; BP, blood pressure; COPD, chronic obstructive pulmonary disease; N/A, not applicable; VEGFI, vascular endothelial growth factor inhibitor.
